# Practical Approaches for Promoting Health Equity in Communities

**DOI:** 10.1007/s10995-022-03456-9

**Published:** 2022-08-03

**Authors:** Larissa Calancie, Ann Batdorf-Barnes, Sarah Verbiest, Nevillene White, Kristen Hassmiller Lich, Giselle Corbie, Amy Mullenix, Dorothy Cilenti

**Affiliations:** 1 ChildObesity180, Friedman School of Nutrition Science and Policy, Tufts University, Medford, USA; 2 Population Health Partners, PLC, Rochester Hills, MI USA; 3 School of Social Work & School of Medicine, University of North Carolina at Chapel Hill, Chapel Hill, USA; 4 New York State Department of Health, Bureau of Women Infant and Adolescent Health, Albany, NY USA; 5 Health Policy and Management Department, Gillings School of Global Public Health, University of North Carolina at Chapel Hill, Chapel Hill, USA; 6 Center for Health Equity Research, Social Medicine Department, School of Medicine, University of North Carolina at Chapel Hill, Chapel Hill, USA; 7 Department of Maternal & Child Health, Gillings School of Global Public Health, National MCH Workforce Development Center, University of North Carolina at Chapel Hill, Chapel Hill, USA

## Abstract

The Maternal and Child Health workforce, public health practitioners, researchers, and other groups need clear, practical guidance on how to promote health equity in the communities they serve. The National Maternal and Child Health Workforce Development Center’s Health Equity Team synthesized eight approaches for promoting health equity that drew on their experience working with public health practitioners and communities. The approaches are to: Expand the understanding of the drivers of health and work across sectors; Take a systems approach; Reflect on your own organization; Follow the lead of communities who experience injustices; Work with community members, decision-makers, and other stakeholders to prioritize action; Foster agency within individuals and collective action within groups; Identify and collect data to show where health inequities currently exist to inform equitable investment of resources; and Be accountable to outcomes that reflect real improvements in people’s lives. The fields of maternal and child health and public health more broadly is already engaged in the complex work of promoting equity and social justice, and in doing so, should refine, challenge, add to, and build upon these approaches.

While there is increasing awareness of health disparities and racial injustices in the United States, serious problems remain, and immediate action is needed. Persistent disparities across the life course, such as a two to five-fold difference in infant mortality rates among Black infants compared to other racial groups and a 2.4 times higher risk of maternal mortality among Black women compared to white women (Singh, 2021), underscore the need for the Maternal and Child Health (MCH) workforce to do much more to promote health equity. Social justice movements like Black Lives Matter and tragic events such as the murder of George Floyd galvanized a large multi-racial response to address systemic racism and other deeply-rooted systems of oppression that give rise to health disparities in the U.S. and around the world (The Lancet, 2020). In 2020, the American Public Health Association sent out a call for action to address racism as a public health emergency (American Public Health Association, 2020). To advance health equity, MCH and public health practitioners must take actions that promote social justice; these include actions that are anti-racist, share power with, and elevate the knowledge, skills, and wisdom of communities of color and other groups experiencing injustices (Paine et al., 2021). Public health practitioners need clear, practical guidance on *how* to promote health equity in the states and communities they serve.

According to the Department of Health and Human Services, health equity is “the attainment of the highest level of health for all people. Achieving health equity requires valuing everyone equally with focused and ongoing societal efforts to address avoidable inequalities, historical and contemporary injustices, and the elimination of health and health care disparities” (Department of Health and Human Services, 2010). Multiple studies and reports point out the need for the MCH Workforce, and the public health workforce more broadly, to become skilled promoters of health equity and social justice (Association of Maternal & Child Health Programs, 2016; Bogaert et al., 2019; Erwin & Brownson, 2017; National Consortium for Public Health Workforce Development, 2017). Health disparities, or differences in health outcomes driven by characteristics associated with discrimination or marginalization, result from systems that consistently advantage some groups and disadvantage others over time (Braveman & Gruskin, 2003). Promoting health equity requires intentional vigilance. Disparities are more appropriately described as “inequities” when they result from injustices that are perpetuated by multiple interlocking systems, such as institutional racism, that have evolved over centuries to predictably produce unfair outcomes (Gallegos & Chilton, 2019; The Center for Assessment and Policy Development and MP Associates and World Trust Educational Services, 2020). Transforming systems that create and perpetuate inequities into systems where everyone can thrive is a powerful goal that the MCH Workforce should continuously work towards with a sense of urgency. The goal implies a commitment to social justice as a value that motivates the field. Achieving this goal will require an ongoing commitment to multi-strategic efforts that address the structural and systematic drivers of racial injustice. It is unreasonable and implausible that one or a few changes at one point in time will achieve equity. While changing systems is challenging, it is not impossible.

This commentary suggests eight approaches for the MCH Workforce, public health practitioners, researchers, and other groups working to promote health equity in communities. Public health is deeply rooted in community-based work (Israel et al., 1998). Building on this foundation, we join others in calling for a shift in power from organizations with financial resources including grantmakers, to communities that experience inequities (Foxwoth & Haymon, 2021). These approaches are not a recipe to follow; they are concepts that can be adopted and tailored to address specific inequities in communities. Examples of specific steps, strategies, actions, and activities that have been implemented in communities are available from organizations such as the Association of Maternal and Child Health Professionals (AMCHP), the nonprofit FSG, and the Robert Wood Johnson Foundation. Best practices for promoting equity are rapidly evolving as researchers and practitioners work to promote equity and share lessons learned.

The approaches presented here emerged from discussions within the National Maternal and Child Health Workforce Development Center’s (MCH Center) Health Equity team and are broadly applicable to public health efforts beyond MCH. The approaches are informed by the team’s experience working with public health practitioners and with communities over the past nine years. During that time, the MCH Center engaged intensively with 48 states to support health transformation in public health systems, in collaboration with cross-sector partners. Members of the health equity team advised on those health transformation efforts as they related to equity and brought their own experiences to bear as community organizers, state health department officials, academics, and public health practitioners. The authors here attempt to synthesize their equity learning in approaches that are actionable for practitioners in the field.

## Approaches for Promoting Health Equity

### Expand the Understanding of the Drivers of Health and Work Across Sectors

Develop a shared understanding within your organizations and in your communities about the many factors beyond health care that shape health and create inequities (Region V Social Determinants of Health Team of the Infant Mortality Collaborative Improvement and Innovation Network [CoIIN] and the Health Resources and Services Administration [HRSA], 2016). Social determinants of health, or the conditions in which people are born, grow, live, work, and age, strongly influence quality and length of life (County Health Rankings, 2014; Marmot et al., 2008). Social determinants are the result of deeply entrenched structural determinants of health, including political, social and economic policy, power imbalances, and cultural influences (e.g., mindset, implicit bias) (Region V Social Determinants of Health Team of the Infant Mortality CoIIN and HRSA, 2016; County Health Rankings, 2022). To initiate and sustain system and structural changes requires partnerships with multisector collaborations that span multiple determinants of health to promote synergy in pursuit of a common goal of achieving health equity (Kumanyika, 2019)—a process that must be grounded in rich understanding of the key interconnected determinants, system structure flaw, and/or problematic mindsets that must be changed.

### Take a Systems Approach

Learn how to use systems thinking to connect themes and patterns about how and why systems advantage some and disadvantage others. Use system models to inspire action and new ideas (Frerichs et al., 2016). If systems thinking is new to your group, provide opportunities to learn and practice these methods for ‘seeing the big picture.’ The MCH Navigator offers five-minute videos describing systems thinking tools frequently used by the Systems Integration Core at the MCH Workforce Development Center (http://www.mchnavigator.org/trainings/systems-integration.php; also see http://systemsintegrationcore.web.unc.edu/). A website titled ‘The Systems Thinker’ has many helpful resources as well (https://thesystemsthinker.com/). Document and share examples of when systems fail communities, such as the negative health effects families’ experience when nutrition benefits are disrupted (de Cuba et al., 2019). Identifying problems within existing systems can uncover opportunities for policy change (Chilton et al., 2009). Promoting policies and systems that enable health for all is an essential public health service (Centers for Disease Control & Prevention, 2021).

### Reflect on Your Own Organization

Organizations that do not support diversity, equity, and inclusion within their own staff and policies are likely to perpetuate imbalances in their community-based work. Build awareness about who makes up your organization, who your organization partners and contracts with, and how decisions are made within your organization. Examine biases and blind spots, and employ strategies for being inclusive, empathetic, and effective leaders in promoting health equity. Working through the Foundational Practices for Health Equity Learning and Action Tool specifically designed for public health organizations (https://www.mchnavigator.org/documents/Foundational-Practices-for-Health-Equity-2018-FINAL.pdf) is an excellent opportunity to examine your own organization through a health equity lens. The tool applies to many of the approaches in this commentary. In addition to the Foundational Practices, organizations may need training from health equity, anti-racism, and workforce development experts as the goal of being anti-racist is a life-long journey. As groups engage in continuous learning and improvement, it is important to identify a ‘historian’ in an organization. This person keeps track of what has been done so that teams keep moving forward and do not forget key steps. The historian helps make connections across all the aspects of a complex endeavor. Be mindful of the identity of this person since their perspective will shape how the process is documented.

### Follow the Lead of Communities Who Experience Injustices

Community members with the lived experience of health inequities should be recognized for their knowledge and expertise. Explore existing initiatives, perceptions, and knowledge about disparities with the community impacted by structural inequities. Listen to community members’ perspective of which systems, policies, and practices might be creating barriers to optimal health, resulting in health inequities. Ask: Do community members have a long and rich history of working to address systemic barriers to health, or is this a new area of concern and action? Do community members already have momentum in addressing other structural inequities (such as in housing or the environment) that can produce broader community synergies to address injustices that are inclusive of local public health concerns? Work to build a strong understanding of community members’ needs and perspectives and let that understanding guide how health equity work is discussed, undertaken, and communicated to outside audiences.

Be mindful of the way disparities are framed. When data are presented in a way that neglects the structures that lead to inequities, it can perpetuate implicit bias, and/or convey that the situation is hopeless. Use a strengths-based approach by building on assets in a community rather than focusing on deficits (Volpe et al., 2019). Culture, class, history, and other contextual factors are strong drivers of health, and they vary throughout communities. Grow your understanding of the community context, including the harm that your organization and/or predominantly white organizations may have caused. Trust is critical in this work. If trust between communities and public health entities has been broken, work carefully to re-build it.

Work to share power with communities of color by supporting and resourcing resident-led efforts and by addressing power imbalances. Value all community members’ cultural, historic, and social knowledge. Listen. Really listen to community members’ perceptions of why a problem exists, potential solutions, and preferences. Build community engagement skills in the MCH Workforce using resources like the Centers for Disease Control and Prevention’s Practitioners’ Guide for Advancing Health Equity (https://www.cdc.gov/nccdphp/dch/pdf/ HealthEquityGuide.pdf) and Principles of Community Engagement (https://www.atsdr.cdc.gov/communityengagement/pdf/PCE_Report_508_FINAL.pdf). Many organizations, such as ReThink Health, Bridging Community to Health, and Health Impact Partners, offer health equity and community engagement guides. Consider applying human-centered design thinking principles at all stages of your work. In human-centered design, people (e.g., community residents, consumers, customers, program participants) are involved in all steps of a problem-solving process, from understanding the problem, to brainstorming, prototyping, and implementing solutions. IDEO and Acumen Plus are a few of many organizations that offer human-centered design resources and trainings.

### Work with Community Members, Decision-Makers, and Other Stakeholders to Prioritize Action

Again, be mindful of power dynamics and really listen to stakeholders. System maps and models can help here too. It may be more productive to have hard conversations and disagree with a model than with each other (Black, 2013; Black & Andersen, 2012; Luna-Reyes et al., 2019; Matson et al., 2021). Make sure your solutions align with community strengths, needs, priorities, and constraints. Map your actions onto an evaluation plan developed with stakeholders. Use evidence-based strategies for promoting equity in MCH when they exist (e.g., http://www.amchp.org/programsandtopics/BestPractices/InnovationStation/Pages/Innovation-Station.aspx and https://www.mchevidence.org/). Be prepared to pivot, change, and realign based on what stakeholders tell you.

### Foster Agency within Individuals and Collective Action Within Groups

Consider who typically leads initiatives in your organization and in the community groups with whom you partner. Encourage those with power to step back and provide support and financial resources so that people who live with inequity, and who have wisdom, know-how, and strong relationships, can step forward and lead. Doing so provides an opportunity for individuals to develop agency and “make purposeful choices” (Kelly & Tamber, 2018), and for groups to take collective action and build social capital within communities. Employ community residents when possible and compensate them fairly.

### Identify and Collect Data to Show Where Health Inequities Currently Exist to Inform Equitable Investment of Resources

Consult the data experts in your organization and in your community to see what data are available. For example, a website called Data You Can Use (https://www.datayoucanuse.org) connects community members in Milwaukee with local professionals who can assist in accessing, interpreting, and communicating neighborhood-level data. As another example, MEASURE offers data tools and services to mobilize communities to eliminate social disparities (https://wemeasure.org). Use systems models for ideas about what to measure and use data to test systems maps and models when possible. When collecting primary data, be sure to use collection methods, such as oversampling, that help identify where inequities exist. Collect data about community assets too. Partner with residents in the community to co-create evaluation plans for programs and initiatives. Collect measures that are meaningful to the community to show whether efforts are making an impact on the processes and outcomes that communities who experience injustices care about. Here are a few more considerations:

#### Work with Communities

Consider adopting community-based participatory research approaches (Wallerstein & Duran, 2006) and empowerment evaluation where possible (Fetterman & Wandersman, 2005). Work with communities to determine what data to collect, and train community members to help collect, analyze, and act using data when possible.

#### Finding Data

Identify many data sources, including quantitative and qualitative data. Example data sources are found on pages 18–19 and Appendix C of CDC’s Practitioners’ Guide for Advancing Health Equity. Be mindful of whose perspective data are being collected from. What assumptions are we making when we ask questions a certain way? Are we gathering information that can lead to actions to address health issues and disparities that are most important to the communities we serve? Have we considered the strengths and assets in communities?

#### Data Quality

Try to ‘ground truth’ data—is the data accurate (Sadd et al., 2014)? How often is it collected? Is the organization or individual collecting data credible? What might be missing from the data? It is being used in context and respectfully so that it does not perpetuate racism and bias?

#### Program and Initiative Evaluation

Identify outcomes of interest to communities and include them in the design, data collection, and data analysis of your evaluation plan. Think about how you can collect data to answer these questions: How can we show that what we are doing is working? How will we know if it is not working? Collect data to determine who was and was not served by a program or initiative, and who stayed in a program and who left out and why. Collect data that demonstrates system change, such as fostering collective agency, building trust between communities of color and public health, greater community engagement, and stronger social networks. Share data and evaluation results with community members when possible. Use accessible language and formats to design engaging dissemination materials (Cooksey Stowers, et al., 2022).

### Be Accountable to Outcomes that Reflect Real Improvements in People’s Lives

Challenge your organization to revisit goals and objectives and ask whether you are achieving them. Ask questions such as: Are we reaching populations that have been previously underserved by public health and healthcare systems? Do we focus on groups that have been historically marginalized through social and economic policy? Are we fostering agency and sharing power with community members? Are gaps in health status decreasing? Are we truly engaging community members to co-create solutions to complex public health challenges? Are we supporting collaboration across sectors? Challenge your organization to align resources with an outcome-oriented approach. Ask: Are we engaging partners in conversation about aligning funding streams and developing sustainable financing mechanisms tied to outcomes? If not, why not? Update your system models to support future work.

#### Summary

In summary, health equity and social justice are vital to a healthy, thriving society. The approaches presented here contribute to on-going conversations about *how* the MCH Workforce and public health practitioners can promote health equity in their communities. The approaches emphasize the importance of sharing power with those who have experienced inequities, centering community expertise, and the role of the MCH Workforce and public health practitioners in supporting community-led initiatives. Your organization is likely already implementing some of these approaches; celebrate your movement and contributions toward a more equitable society. Then, challenge your organization to adopt additional approaches that move the needle even further. At the individual level, training, practice, on-going improvement, humility, and self-reflection are all critical for developing the skills and mindsets needed for improving health outcomes for communities of color, under-resourced communities, and other groups experiencing disadvantage. Moving beyond individuals, organizational, policy, and system changes will be needed to facilitate the uptake of some suggested approaches. The fields of MCH and public health must continue to engage in the complex work of promoting equity and social justice, and in doing so, should refine, challenge, add to, and build upon these approaches.

## Data Availability

Not applicable.
